# Risk Factors of Muscle Wasting in Women with Rheumatoid Arthritis: Relevance of the Persistent Failure of Conventional Combination Therapy

**DOI:** 10.3390/healthcare10102004

**Published:** 2022-10-11

**Authors:** Eli E. Gomez-Ramirez, Melissa Ramirez-Villafaña, Jorge I. Gamez-Nava, Fidencio Cons-Molina, Norma A. Rodriguez Jimenez, Ana M. Saldaña-Cruz, Ernesto G. Cardona-Muñoz, Sylvia E. Totsuka-Sutto, Juan M. Ponce-Guarneros, Xochitl Trujillo, Miguel Huerta, Alfonso J. Cruz-Jentoft, Laura Gonzalez-Lopez

**Affiliations:** 1 Doctorado en Farmacología, Centro Universitario de Ciencias de la Salud, Universidad de Guadalajara, Guadalajara 44340, Mexico; 2 Research Group for the Assessment of Prognosis Biomarkers in Autoimmune Disorders, Guadalajara 44340, Mexico; 3 Departamento de Fisiología, Centro Universitario de Ciencias de la Salud, Universidad de Guadalajara, Guadalajara 44340, Mexico; 4 Doctorado en Salud Publica, Departamento de Salud Pública, Centro Universitario de Ciencias de la Salud, Universidad de Guadalajara, Guadalajara 44340, Mexico; 5 Centro de Investigación de Artritis y Osteoporosis, Mexicali 21200, Mexico; 6 Unidad de Medicina Familiar 97, Instituto Mexicano del Seguro Social (IMSS), Magdalena 46474, Mexico; 7 Centro Universitario de Investigaciones Biomédicas (CUIB), Universidad de Colima, Colima 28040, Mexico; 8 Servicio de Geriatría, Hospital Universitario Ramón y Cajal (IRYCIS), 28034 Madrid, Spain

**Keywords:** rheumatoid arthritis, muscle wasting, myopenia, therapy failure, csDMARDs

## Abstract

**Background:** Muscle wasting, also known as myopenia, is frequent in rheumatoid arthritis (RA). To date, it is still unknown if the failure of pharmacologic therapies increases the risk of myopenia in RA. **Objective:** To identify if treatment failure with conventional synthetic DMARDs (csDMARDs) constitutes an independent risk factor of muscle wasting in women with RA. **Methods:** This was a cross-sectional study. We included 277 women with RA. Assessments in RA patients included: clinical, epidemiological, and therapeutic variables. The skeletal muscle index (SMI) was estimated by DXA, and myopenia was diagnosed if they had an SMI < 5.45 kg/m^2^. Multivariable logistic regression models identified risk factors of myopenia. **Results:** Muscle wasting was observed in 28.2% of patients with RA. The risk factors of myopenia in RA were menopausal (OR: 4.45, 95% CI: 1.86 to 10.64) and failure of combined therapy with csDMARDs (OR: 2.42, 95% CI: 1.15 to 5.07). The increased body mass index was protective (OR:0.81, 95% CI: 0.75 to 0.87). **Conclusions:** Around one of four patients with RA presented muscle wasting. Muscle wasting is related to treatment failure of combined csDMARDs; other factors influencing the presence of muscle wasting is being postmenopausal, whereas, the body mass index was a protective factor.

## 1. Introduction

A low skeletal muscle mass (myopenia) is listed in the diagnostic criteria of many conditions, including malnutrition, cachexia, and sarcopenia [1,2,3]. Patients with inflammatory rheumatic diseases have a higher frequency of low skeletal muscle mass. In rheumatoid arthritis (RA) patients, the presence of myopenia is relevant for studies of sarcopenia or cachexia [4,5].

There is a broad variation in the prevalence of myopenia across the studies performed in RA patients, depending of a multiplicity of factors, including race; however, studies have shown a high frequency of low skeletal muscle mass compared with their controls [6,7,8,9,10], the prevalence rates of myopenia in RA vary widely from 11% to 49% [11,12]. A deteriorated skeletal muscle mass should be detected early and treated adequately in RA patients because low muscle mass may be associated with muscle weakness, impairment of functioning, and deteriorated health-related quality of life, among other adverse consequences [5,13,14].

The wasting of muscle has been identified in patients with RA associated to the persistence of active disease that is induced by several factors, such as an increase in pro-inflammatory cytokines (IL-6, TNF-α, and IL-1β), elevated acute-phase reactant levels, and reduced physical activity; as consequence of these inflammatory processes, an increased muscle catabolism is developed [12,13,14]. With these data, it can be hypothesized that a failure to achieve an adequate therapeutic response could be a major risk factor for deteriorated muscle mass. Nevertheless, to date, there is only limited and inconsistent information about the relation between myopenia and the type of drugs used for the treatment in RA [6,8,10,12,15,16], but none of them assessed combined therapy with conventional synthetic disease-modifying antirheumatic drugs (csDMARDs). Treatment with csDMARDs is considered the first line of treatment in RA patients and should be prescribed early [17,18]. Combination therapy with csDMARDs is a well-recognized therapeutic strategy after the failure of monotherapy, and the combination of csDMARDs has demonstrated higher effectivity than monotherapy with one DMARD [17,19]. In developing countries, where many RA patients have economic restrictions to follow a subsequent therapy with biologic agents, the use of combined therapy with csDMARDs constitutes the most frequent therapeutic strategy [20]. Failure of combined therapy with csDMARDs is associated with worsening of the disease, radiological damage progression, and a high risk of permanent work disability [21,22].

To date, there is a lack of information on whether the failure of combined therapy with csDMARDs is associated with myopenia in patients with RA. Therefore, this study had the aim of to identify in a multivariable approach if treatment failure with conventional synthetic DMARDs (csDMARDs) constitutes an independent risk factor of muscle wasting in women with RA.

## 2. Methods

This cross-sectional study was performed in 277 Mexican women with RA who were recruited by invitation in an outpatient research clinic of chronic diseases at the University of Guadalajara (Instituto de Terapeutica Experimental y Clinica (INTEC), Centro Universitario de Ciencias de la Salud, Universidad de Guadalajara) in Guadalajara city, Mexico. This study was performed by researchers of the Group for the Assessment of Prognosis Biomarkers in Autoimmune Disorders. The characteristics and members of this group had been published elsewhere [23]. Patients were eligible to participate if they met the 1987 American College of Rheumatology (ACR) criteria for RA, aged ≥18 years.

We excluded pregnant or breastfeeding patients and those with the presence of other autoimmune diseases. Additionally, we excluded patients with overlapping syndromes, infections (including acute infections, chronic viral infections such as hepatitis B or C, human immunodeficiency virus, and tuberculosis). Other exclusion criteria were active cancer, hypothyroidism, chronic renal failure (defined as a serum creatinine > 1.2 mg/dL and an estimated glomerular filtration rate lower than 50 mL/min), and an increase in transaminase levels (greater than two-fold of normal values).

All the RA patients invited to participate in the study signed a voluntary informed consent form.

### 2.1. Study Protocol

RA patients were interviewed and clinically examined by trained researchers, who performed a chart review assessing epidemiological and clinical characteristics. Body mass index (BMI) was calculated in kg/m^2^ and classified into two categories: (a) low and normal weight (≤24.9 kg/m^2^) and (b) overweight and obesity (≥25 kg/m^2^). 

### 2.2. Clinical Assessment 

The DAS28-ESR index was used to classify RA patients into two groups: (a) active disease (DAS28-ESR ≥ 2.6) and (b) in remission of the disease (DAS28-ESR score of <2.6) [24,25]. Physical functioning was investigated with the Spanish version of Health Assessment Questionnaire-Disability Index (HAQ-DI).

### 2.3. Body Composition Measurements

Body composition measurement was assessed by dual-energy X-ray absorptiometry (DXA) (LUNAR 2000, Prodigy Advance; General Electric™, Madison, WI, USA). Fat mass (%) and skeletal muscle mass were estimated using this method. Skeletal muscle index (SMI) was computed according to Baumgartner et al. [26]: skeletal muscle mass of the extremities (SMME) was first computed as the sum of the muscle mass of the four limbs obtained from DXA results, and then SMME was adjusted by height (meters squared) (SMI = SMME/height^2^) [12]. The result of this computation gives the SMI value in kg/m^2^. A low SMI in women is considered if the result is <5.45 kg/m^2^ [26]. 

### 2.4. Definition of Study Groups

After the assessment of SMI, we grouped RA patients as: (a) RA with muscle wasting (SMI < 5.45 kg/m^2^) and (b) RA with normal skeletal muscle mass (SMI ≥ 5.45 kg/m^2^). These groups were used for the assessment of risk factors comparison.

### 2.5. Pharmacological Treatment

In our clinical setting, once patients are diagnosed with RA by a rheumatologist, the prescription of monotherapy with a csDMARDs is considered as the first line of treatment, methotrexate being the drug most used in monotherapy, unless there was a contraindication; in that case, other csDMARDs such as sulfasalazine, antimalarials, leflunomide, or azathioprine were initiated based on patient preferences and the rheumatologist’s clinical judgement. After 3–4 months of monotherapy, patients were re-assessed, and according to the rheumatologist’s judgement of achieved a therapeutic response and the presence of side effects, the rheumatologists could recommend to continue with the initial csDMARDs or use one of the following strategies: (a) change to a monotherapy with a second csDMARDs, (b) start a biologic-DMARD (in our settings, this strategy is less frequently used because of economic issues), or (c) use the step-up strategy of combined therapy with two or more csDMARDs. The step-up approach with csDMARDs is the therapeutic strategy most commonly used in our setting after failure of monotherapy. The use of biologic-DMARDs is planned considering economic restrictions, taking into account the patients’ decision and economic feasibility.

### 2.6. Definition of Failure of Combined Therapy with csDMARDs

For patients who started a combined therapy with csDMARDs (unless side effects developed), this therapy was maintained for at least 3 to 4 months using recommended doses. A first failure of combined therapy with csDMARDs was considered following the EULAR criteria of poor response if the patients maintained a DAS28-ESR > 3.2 [27]. According to the treat-to target guidelines, these patients did not achieve remission or low-disease activity. In RA patients with a first failure of combined therapy, a second approach using a different combination of csDMARDs was used. Definite failure of combined therapy with csDMARDs was considered when the patients did not achieve remission or at least low-disease activity with a second different combined therapy with csDMARDs. 

### 2.7. Laboratory Determinations of Rheumatoid Factor and Acute-Phase Reactants

After an eight-hour fast, a sample of peripheral venous blood was obtained on the same day of the assessment from each patient in all patients. Serum was stored at −20 °C. Rheumatoid factor (RF) was measured by nephelometry, erythrocyte sedimentation rate (ESR) was measured by Westergren method, and C-reactive protein (CRP) was quantified by nephelometry.

### 2.8. Statistical Analysis

Quantitative variables were described as the means and standard deviations (SD), and qualitative variables were described as frequencies and percentages (%). Comparisons of proportions between groups (RA with muscle wasting vs. RA with normal skeletal muscle mass) were made using the Chi-square test (or Fisher exact test, when required), whereas comparisons of means between two groups were computed using Student’s *t*-test. We performed multivariate logistic regression analyses to identify risk factors of muscle wasting (dependent variable) while adjusting for potential confounders. Covariates introduced in the models were variables considered with biological plausibility for influencing this dependent variable. We utilized the forward stepwise method. Statistical significance was set at the *p* ≤ 0.05 level. All statistical analyses were performed using R statistical software version 4.0.2 [28].

### 2.9. Ethics

The study protocol was performed following the lineaments of the Helsinki Declaration. Prior to participation, all the participants signed a voluntary informed consent form. All the procedures involved in this study had the approval of the Research and Ethics Committee of the University Center (Instituto de Terapeutica Experimental y Clinica, Centro Universitario de Ciencias de la Salud, Universidad de Guadalajara). Code of approval: CEI/499/2019.

## 3. Results

A total of 277 women with RA were included. Table 1 shows the clinical characteristics of the RA patients. The mean age was 57.4 years, and the disease duration was 12.9 years. Most of patients had menopause (79.1%), and hormone replacement therapy was received by 34.7%. Of the 277 RA patients, 97.5% had taken the prescription of synthetic DMARDS (2.5% were not compliant to treatment), and 15.9% had repeated failure of combined therapy with csDMARDs and were arranged to start treatment with a biologic-DMARD (Table 1).

Table 2 compares the clinical characteristics in women RA patients with muscle wasting and RA with normal skeletal muscle mass. RA + muscle wasting had lower frequency of overweight/obesity (*p* < 0.001) and lower frequency of hormone replacement therapy (*p* = 0.01). Instead, these patients had higher frequency of menopause (*p* = 0.006) and of failure of treatment to combined synthetic DMARDs. The mean SMI was higher in RA patients receiving hormone replacement therapy compared with those without this therapy (6.27 ± 1.18 vs. 5.90 ± 1.09, *p* = 0.009), (data are not shown in table).

Table 3 describes the risk factors associated with muscle wasting in 277 RA patients. After adjustment for age, smoking, alcohol consumption, sedentary lifestyle, hormone replacement therapy, disease duration, disease activity, functional disability, corticosteroid dosage, and diabetes mellitus. The risk factors of muscle wasting in RA were menopause (OR: 4.45, 95% CI: 1.86 to 10.64) and failure of combined therapy with csDMARDs (OR: 2.42, 95% CI: 1.15 to 5.07). The body mass index was a protective factor of muscle wasting (OR:0.81, 95% CI: 0.75 to 0.87).

## 4. Discussion

In the present study, we found that 78 out of 277 women with RA had muscle wasting. The presence of a failure of combined treatment with csDMARDs and menopause were the main risk factors of muscle wasting. Instead, a normal or increased BMI is a protective factor of myopenia.

Myopenia in RA was identified in our study in 28.2%; a similar prevalence was observed in studies performed in the United States (Giles et al.), Turkey (Alkan et al.), and France (Tournadre et al.), in which the prevalence of myopenia varied from 20% to 28.6% [6,7,10]. However, we observed lower prevalence of myopenia in RA compared with Torii et al. [12] (49% in Japan); Ngeuleu et al. [13] (39.8% in Morocco); and Dogan et al. [8] (43.3% in Turkey). The lower prevalence of myopenia in RA was reported in two studies, one of which was performed in Spain by Delgado-Frias et al. [9] (13%) and the second study by Baker et al. [11] in the United States (11%). Such variations can be attributable to differences in the population of RA patients being included, such as differences in race, genetic predisposition, distinct disease duration, severity of the disease, concurrent comorbid diseases, and non-similarity of the therapeutic strategies [6,9,12,13]. Therefore, these studies have multiple factors that can influence the presence of muscle wasting. These findings should alert physicians to detect myopenia earlier in RA patients, stabilizing strategies directed at avoiding the consequences of this complication.

Both the presence of menopause and the failure of combined therapy with csDMARDs increased the risk muscle wasting in RA patients. To our best knowledge, the finding that these two features combined increased the risk of myopenia has not been reported previously using a multivariable approach.

We were unable to evaluate the effect of the lack of treatment with DMARDs because all our patients received these drugs and only a very small proportion of RA patients were not compliant to the prescription. Our findings that the lack of a response to combined therapy with csDMARDs is a risk factor of muscle wasting in RA, is consistent with the evidence that persistent inflammatory disease activity, a decrease in physical performance, and the overexpression of pro-inflammatory cytokines, adipokines, and other molecules increase muscle degradation and can lead to the development of low muscle mass in these patients [5,14,29]. We consider our findings that a repetitive failure of combined treatment with csDMARDs is associated with a greater risk of muscle wasting in RA women to be relevant for clinical care in RA patients. Therefore, in RA with high-risk of myopenia, more effective therapeutic strategies should be considered earlier. Some studies have identified that biological agents such as anti-TNF agents or tocilizumab could have a protective effect on skeletal muscle mass [10,15]. Due to their potent effects in suppressing inflammation by inhibiting cytokines such as TNF-α and IL-6, biologic agents might reduce the catabolic effects of these molecules [10,30]. The findings in the univariable analysis that the use of hormone replacement therapy in menopause RA patients was associated with a lower rate of muscle wasting is relevant because it can encourage clinicians to use a reasoned prescription of hormone replacement therapy to avoid the consequences of a decrease in estrogens on the skeletal muscle. Some authors have identified certain benefits of estrogen therapy on muscle mass gain in postmenopausal women [31,32]. However, in a meta-analysis by Javed AA et al. 2019, the treatment with hormone replacement therapy did not show a significant change in skeletal muscle mass [33].

Although the effect of hormone replacement therapy on the muscle gain in non-rheumatic patients is controverted, the treatment with these hormones has shown to decrease the inflammation in the synovial joints on patients with RA, as well as the erythrocyte sedimentation rate (ESR) and the Disease Activity Score 28 (DAS28) [34]. Probably, hormone replacement therapy could influence muscle mass preservation through its contribution to reducing disease activity. Another hypothesis is the effect of that therapy inhibiting the increased muscle catabolism observed in menopausal women.

### Study Limitations

The first limitation of this study was that our results can be generalized only to females with RA; we did not include in this study men with arthritis. New studies including males with this disease should be performed in order to identify the risk factors in this subpopulation. 

An evident limitation of our study is that only a small number of patients with RA received biological therapies and that patients received these treatments later on in their disease evolution. Biologic agents are usually not considered in our center as the first line of treatment; these agents are prescribed after the failure of two or three csDMARDs in combination therapy. Therefore, this study did not include a group of RA patients with an early onset of treatment with biologics. A future cohort comparing RA patients treated early with biologics versus treated with combined therapy with csDMARDs at a similar point of disease duration is required to identify if biological agents can prevent the development of wasting muscle.

Other limitations of our study should be noted. We did not measure nutritional status, and malnutrition is a precipitant factor for muscle deterioration. Future studies should include the analysis of nutritional status as a relevant factor in these patients with RA. In addition, this study focused on the measurement of muscle mass, but a complete assessment of muscle function, muscular strength, and assessment of nutrition in conjunction with muscle mass is relevant for a more complete assessment of sarcopenia [3,5].

## 5. Conclusions

Muscle wasting was observed in 28.2% of Mexican women with RA participating in this study. Continued failure of combined csDMARDs and menopause are a strong risk factors for muscle wasting in RA, independent of traditional risk factors. An early detection of therapy failure of combined csDMARDs should be followed by considering other treatments and measures could contribute to avoiding the negative effects of this therapy failure on muscle wasting or myopenia in RA patients and their consequences. Further long-term studies and follow-ups are required to identify the most valuable measures to ameliorate muscle wasting, thus decreasing the impact of myopenia in these patients with RA.

## Figures and Tables

**Table 1:** Clinical characteristics in the patients with Rheumatoid Arthritis including in the study.

Variables	n = 277
Age, years	57.4 ± 10.1
Menopause, n (%)	219 (79.1)
Hormone replacement therapy, n (%)	96 (34.7)
Disease duration, years	12.9 ± 9.6
HAQ-DI score, units	0.57 ± 0.5
Functional disability (HAQ-DI ≥ 0.60), n (%)	128 (46.2)
DAS28 score, units	3.7 ± 1.5
-Remission (DAS28-ESR < 2.6);	73 (26.4)
-Low (DAS28-ESR 2.6–3.1)	51 (18.4)
-Moderate (DAS28-ESR 3.2–5.1)	104 (37.5)
-Severe disease activity (DAS28-ESR > 5.1).	49 (17.7)
Corticosteroids use, n (%)	239 (86.3)
Corticosteroids dose, mg/day	4.9 ± 2.8
Corticosteroids dose/Disease duration ratio, mg/day/years	64.9 ± 70.9
Synthetic-DMARD, n (%)	270 (97.5)
-Methotrexate, n (%)	167 (60.3)
-Leflunomide, n (%)	99 (35.7)
-Sulfasalazine, n (%)	88 (31.8)
-Azathioprine, n (%)	43 (15.5)
-Chloroquine, n (%)	41 (14.8)
Treatment failure with combined csDMARDs, n (%)	44 (15.9)
Laboratory variables	
CRP, mg/L	17.1 ± 23.7
ESR, mm/Hr	26.4 ± 12.5
RF, UI/mL	157 ± 365.7
**Body composition and skeletal muscle mass measurement**	
Body mass Index, kg/m^2^	27.8 ± 4.2
-Overweight and obesity, n (%)	203 (73.3)
Skeletal Muscle Index (SMI), n (%)	6.03 ± 1.1
-Muscle wasting, n (%)	78 (28.2)

Quantitative variables are expressed in means ± SD and qualitative variables in frequency (%), DAS28: Disease Activity Score of 28 joints, HAQ-DI: Health Assessment Questionnaire-Disability Index, csDMARDs: Conventional synthetic disease-modifying antirheumatic drugs, CRP: C-reactive protein, ESR: erythrocyte sedimentation rate.

**Table 2:** Comparison of clinical characteristics in women with Rheumatoid Arthritis with normal vs. low-skeletal muscle mass.

Variables	RA Normal Skeletal Muscle Mass (SMI ≥ 5.45) n = 199	RA + Muscle Wasting (SMI < 5.45) n = 78	*p*
Age, years	56.9 ± 10.8	58.6 ± 8.1	0.17
Age ≥ 50 years old, n (%)	152 (76.4)	69 (88.5)	**0.02**
Alcohol consumption, n (%)	12 (6)	4 (5.1)	0.77
Smoking, n (%)	18 (9)	5 (6.4)	0.47
Sedentary lifestyle, n (%)	135 (67.8)	52 (66.7)	0.85
Menopausal, n (%)	149 (74.9)	70 (89.7)	**0.006**
Hormone replacement therapy, n (%)	78 (39.2)	18 (23.1)	**0.01**
Weight, kg	68.8 ± 10.4	62.3 ± 11.8	**<0.001**
Body mass Index, kg/m^2^	28.5 ± 4.1	25.8 ± 3.9	**<0.001**
-Overweight or obesity, n (%)	163 (81.9)	40 (51.3)	**<0.001**
Hypertension, n (%)	78 (39.2)	29 (37.2)	0.75
Diabetes Mellitus, n (%)	22 (11.1)	3 (3.8)	0.06
**Clinical characteristics**			
Disease duration, years	13.03 ± 9.8	12.5 ± 9.1	0.67
HAQ-DI score, units	0.58 ± 0.5	0.53 ± 0.5	0.51
Functional disability, n (%)	93 (46.7)	35 (45.5)	0.85
DAS28 score, units	3.7 ± 1.5	3.7 ± 1.4	0.94
Disease Activity (DAS28 > 2.6), n (%)	147 (73.9)	57 (73.1)	0.89
Corticosteroids use, n (%)	171 (85.9)	68 (87.2)	0.78
Corticosteroids dose, mg/day	4.9 ± 2.9	5.1 ± 2.6	0.54
Synthetic DMARD, n (%)	193 (97)	77 (98.7)	0.67
Failure of combined csDMARDs, n (%)	26 (13.1)	18 (23.1)	**0.04**
**Laboratory findings**			
Positive CRP (≥10 mg/L), n (%)	100 (50.3)	40 (51.3)	0.87
Elevated ESR (≥20 mm/Hr), n (%)	116 (58.3)	47 (60.3)	0.76
Positive RF (≥12 UI/mL), n (%)	124 (62.3)	58 (74.4)	0.06

Quantitative variables are expressed in means ± SD and qualitative variables in frequencies (%). Abbreviations: SMI: Skeletal Muscle Index, DAS28: Disease Activity Score of 28 joints, HAQ-DI: Health Assessment Questionnaire-Disability Index: CRP: C-reactive protein ESR: Erythrocyte sedimentation rate: RF: Rheumatoid factor. Comparison of qualitative variables performed with Chi-square. Comparison of quantitative variables made with Student *t* test. statistical significance *p* < 0.05.

**Table 3:** Multivariable Logistic Regression analysis evaluating factors associated with muscle wasting in women with Rheumatoid Arthritis.

Risk Factors of Low SMI in RA (n = 277)	Forward Method (Stepwise)		
	OR	95% CI	*p*
Menopause	4.45	(1.86 to 10.64)	**0.001**
Treatment failure with combined csDMARDs	2.42	(1.15 to 5.07)	**0.020**
Body mass index	0.81	(0.75 to 0.87)	**<0.001**
Age (years)	Not in the model	-	-
Alcohol consumption	Not in the model	-	-
Smoking	Not in the model	-	-
Sedentary lifestyle	Not in the model	-	-
Hormone replacement therapy	Not in the model	-	-
Diabetes Mellitus	Not in the model	-	-
Disease duration (years)	Not in the model	-	-
Corticosteroids dose (mg/day)	Not in the model	-	-
Disease activity	Not in the model	-	-
Functional disability	Not in the model	-	-

Multivariate analysis: Logistic regression model. forward method stepwise Confidence Interval. Model with dependent variable: low skeletal muscle mass (SMI < 5.45). Excluded covariate variables from the model: Age (years), alcohol consumption, Current smoking, sedentary lifestyle, Hormone replacement therapy, Diabetes Mellitus, Disease duration, Corticosteroids dose, Disease Activity and functional disability. OR, Odds Ratio; 95% CI, 95% statistical significance *p* < 0.05.

## Data Availability

The dataset supporting the conclusions presented in this article is available on request from the corresponding author on reasonable request.
